# Prevalence and determinants of stillbirth in Nigerian referral hospitals: a multicentre study

**DOI:** 10.1186/s12884-019-2682-z

**Published:** 2019-12-30

**Authors:** Friday E. Okonofua, Lorretta Favour C. Ntoimo, Rosemary Ogu, Hadiza Galadanci, Gana Mohammed, Durodola Adetoye, Eghe Abe, Ola Okike, Kingsley Agholor, Rukiyat Abdus-salam, Abdullahi Randawa

**Affiliations:** 1 Women’s Health and Action Research Centre/WHO Implementation Research Group, Benin City, Nigeria; 2 Women’s Health and Action Research Centre, and Vice-Chancellor, University of Medical Sciences, Ondo City, Ondo State Nigeria; 3 Centre of Excellence in Reproductive Health Innovation, University of Benin, Benin City, Edo State Nigeria; 4 Benin City, Edo State Nigeria; 5 Department of Demography and Social Statistics, Federal University Oye-Ekiti, Oye-Ekiti, Ekiti State Nigeria; 6 Department of Obstetrics and Gynaecology, University of Port Harcourt, Port Harcourt, Rivers State Nigeria; 7 Aminu Kano Teaching Hospital, Kano, Kano State Nigeria; 8 General Hospital, Niger State, Minna, Nigeria; 9 General Hospital, Ijaye Abeokuta, Ogun State Nigeria; 10 Central Hospital, Benin City, Edo State Nigeria; 11 Karshi General Hospital, Federal Capital Territory, Abuja, Nigeria; 12 Central Hospital, Warri, Delta State Nigeria; 13 Adeoyo Maternity Hospital, Ibadan, Oyo State Nigeria; 14 Ahmadu Bello University Teaching Hospital, Zaria, Kaduna State Nigeria

**Keywords:** Stillbirth, Stillbirth rate, Referral hospitals, Caesarean section, Nigerian women

## Abstract

**Background:**

In 2015, Nigeria’s estimated 317,700 stillbirths accounted for 12.2% of the 2.6 million estimated global stillbirths. This suggests that Nigeria still makes substantial contribution to the global burden of stillbirths. This study was conducted to determine the prevalence and identify the causes and factors associated with stillbirth in eight referral hospitals in Nigeria.

**Methods:**

This was a cross-sectional study of all deliveries over a period of 6 months in six general hospitals (4 in the south and 2 in the north), and two teaching hospitals (both in the north) in Nigeria. The study population was women delivering in the hospitals during the study period. A pre-tested study protocol was used to obtain clinical data on pregnancies, live births and stillbirths in the hospitals over a 6 months period. Data were analyzed centrally using univariate, bivariate and multivariate logistic regression analyses. The main outcome measure was **s**tillbirth rate in the hospitals (individually and overall).

**Results:**

There were 4416 single births and 175 stillbirths, and a mean stillbirth rate of 39.6 per 1000 births (range: 12.7 to 67.3/1000 births) in the hospitals. Antepartum (macerated) constituted 22.3% of the stillbirths; 47.4% were intrapartum (fresh stillbirths); while 30.3% was unclassified. Acute hypoxia accounted for 32.6% of the stillbirths. Other causes were maternal hypertensive disease (6.9%), and intrapartum unexplained (5.7%) among others. After adjusting for confounding variables, significant predictors of stillbirth were referral status, parity, past experience of stillbirth, birth weight, gestational age at delivery and mode of delivery.

**Conclusion:**

We conclude that the rate of stillbirth is high in Nigeria’s referral hospitals largely because of patients’ related factors and the high rates of pregnancy complications. Efforts to address these factors through improved patients’ education and emergency obstetric care would reduce the rate of stillbirth in the country.

**Trial registration:**

Trial Registration Number NCTR91540209.

Nigeria Clinical Trials Registry. http://www.nctr.nhrec.net/

Registered April 14th 2016.

## Background

Available data indicate that Nigeria has the second highest rate of stillbirth in the world [1]. While the global rate of stillbirth was estimated in 2015 to be 18.4 per 1000 births, with a nearly 25.5% decline between 2009 and 2015, Nigeria experienced a stillbirth rate of 41.67 per 1000 in 2009, with no appreciable improvement in 2015 [2]. In 2015, Nigeria’s estimated 317,700 stillbirths accounted for 12.2% of the 2.6 million estimated global stillbirths [3]. This suggests that Nigeria still makes substantial contribution to the global burden of stillbirths.

Despite its high prevalence, there is persisting lack of accurate data on the risk factors for stillbirths in Nigeria to enable the design of appropriate interventions. While much emphasis is being given to reducing the equally high rate of maternal mortality in the country, very limited efforts have been concentrated on reducing the rate of stillbirth. This is possibly due to the non-inclusion of rates of stillbirth in measuring the achievement of the Millennium Development Goals and now the Sustainable Development Goals, to which the country is devoting priority policy attention. At the global level, it has also been recognized that it is important to make stillbirth visible, recognized and counted [2].

A large proportion of births (over 60%) occur outside health facilities in Nigeria, in the homes of traditional and faith-based birth attendants [4]. Unfortunately, stillbirths occurring in such settings are difficult to capture in data collection systems, especially when traditional norms do not allow the classification of babies that show no signs of life at birth [5]. While household surveys may give better insights on community prevalence of stillbirth, they are limited in providing information on the clinical and health components needed to design health systems interventions for averting future occurrences.

Hospital studies across various regions in Nigeria have reported high rates of stillbirth ranging from a low of 39.9 per 1000 births to a high of 180 per 1000 births [5–8]. Studies also indicate that possible risk factors for stillbirths include multiparity, lack of antenatal care, illiteracy, and mode of delivery [9, 10]. In particular, the large burden of obstetric complications – obstructed labour, pregnancy induced hypertension and anaemia – were reported as making significant contributions to increasing the likelihood of stillbirths in these hospitals.

While single hospital studies provide substantial insights into the nature of the problem, they are limited in their usefulness for designing interventions at the health systems level for addressing the problem. It is within this context that we undertook a multicentre study of rates and determinants of stillbirths in eight selected referral hospitals from four out of the six geo-political zones of the country. We believe that this approach will not only help the comparisons of the rates of stillbirth between the different regions of the country, it will also help the true assessment of the social and other determinants, and the design of systemic interventions needed to address the risk factors associated with stillbirths.

## Methods

### Data source and sample size

The study was conducted in 8 referral hospitals in Nigeria as part of a baseline study for an intervention research on improving the quality of emergency obstetric care for preventing maternal and perinatal mortality in Nigeria.

Nigeria operates a three-tier health care system that comprises of primary health care (PHC) as the entry point, secondary care (regional/general hospitals) as the first referral level and tertiary care (teaching hospitals) as the second level of referral. The PHC facilities provide basic obstetric care, while women with complications are referred to secondary and tertiary care facilities which provide comprehensive emergency obstetric care [11]. This structure of the country’s health system ensures that most fetal deaths in hospitals occur and are recorded in the referral facilities rather than in the PHC facilities. Also, previous studies in Nigeria report under-utilization of PHC facilities for pregnancy care, with most women seeking comprehensive emergency obstetric care directly in referral facilities [12–15]. This situation explains why this study is limited to referral hospitals in four of six regions in Nigeria.

Data were collected from six General Hospitals: 4 in the south and 2 in the north, and two teaching hospitals (both in the north) in Nigeria. The two tertiary hospitals were selected from the Northwest: Ahmadu Bello University Teaching Hospital, Kaduna and Aminu Kano Teaching Hospital Kano. By contrast, the secondary care facilities were selected from three other zones as follows: General Hospital, Ijaye, Abeokuta and Adeoyo Maternity Hospital in the Southwest; General Hospital, Minna, Niger State and Karshi Hospital, Abuja in the North-central, and Central Hospital, Benin City and Central Hospital, Warri from the South South region. We were unable to obtain data from the North-east region because of the ongoing insurgency in the region. Therefore, to ensure equal representation of hospitals between the northern and southern parts of the country, we excluded hospitals in the south-east region from the study.

Data were abstracted from the records of 5262 deliveries in the hospitals during a period of six months (January to June 2014). Information extracted from the case notes included maternal age, employment status, education, place of residence, marital status, antenatal booking, referral status, parity, as well as clinical presentation and the history of current and past obstetric outcomes. Data on the infants included birth weight, gestation age at delivery, mode of delivery, and whether the birth was single or multiple. We particularly documented the total number of deliveries and live births and fresh stillbirths as well as the total number of all fetal deaths before complete expulsion from the mother. A baby born with no signs of life at or after 28 weeks gestation was recorded as stillbirth, according to the World Health Organization (WHO) definition [16]. Fresh and macerated stillbirths were recorded. Births below gestational age 28 weeks and those with missing information on their outcomes were excluded from the analysis. Twin births were also excluded from this analysis because of the difference in the risk of fetal deaths for singleton and multiple births. Thus, this study was limited to a total of 4416 single births.

### Variables and measures

The outcome variable was stillbirth. All delivery outcomes were categorized into two: stillbirths [1] and live births (0). The independent variables were maternal and fetal characteristics comprising of maternal age, grouped into <=19, 20–24, 25–29, 30–34 and 35–49; mothers aged 35–49 years were merged because they were not many in the five-year categories. Other maternal characteristics were employment status, place of residence (urban versus rural), marital status (married and single mothers comprising the never married, widowed, divorced and separated), antenatal booking status, whether the mother was referred or not, number of deliveries (parity), experience of complications such as postpartum hemorrhage, eclampsia or obstructed labour in previous pregnancies, and number of past stillbirths. Fetal characteristics were birth weight, gestational age at delivery (< 37 weeks as preterm and > =37 weeks as term), and mode of delivery comprising normal vaginal, caesarean section, and others (ventuose, forceps, vaginal breech and others). Maternal education was excluded due to a large number of missing cases in the files.

### Analytical approach

Data were analysed using Stata 12 for windows. The distribution of all births and stillbirths by maternal and fetal characteristics was presented using absolute numbers and their corresponding percentages. Characteristics of mothers and the births were presented using percentages. Stillbirth rate was presented as the number of stillbirths expressed as a proportion of total births multiplied by 1000. The causes of stillbirth were presented following a classification of causes of stillbirth by time of death provided in the provisional classification system for global estimates of cause of stillbirths by the Child Health Epidemiology Reference Group (CHERG, Global Alliance for Prevention of Prematurity and Stillbirths (GAPPS) and Saving New-born Lives/Save the Children for WHO (Lawan et al., 2009; Yakoob).

To examine the association between stillbirth and the selected predictors, bivariate and multivariate models of logistic regression were estimated. The bivariate model was unadjusted with each independent variable and the outcome. Inclusion of variables in the adjusted model was based on statistical significance at *p*-value < 0.10 in the bivariate model. .

## Results

### Stillbirth rates

The total number of births, stillbirths and stillbirth rates are presented in Table 1. Out of 4416 single births, 4241 were live births and 175 were stillbirths. The mean stillbirth rate for the 8 facilities within the period was 39.6 per 1000 births. The highest rate was in General Hospital, Minna (67.3/1000 births) whereas the lowest was 12.7/1000 in Karshi General Hospital, Abuja. Four out of the eight hospitals recorded stillbirth rates above the average for the 8 facilities.

**Table 1:** Total Births and Stillbirths by Facility

Facility	Births N(%)	Stillbirth N (%)	Stillbirth Rate (per 1000)
Aminu Kano Teaching Hospital, Kano	778 (17.6)	28 (16.0)	36.0
Karshi General Hospital, Abuja	237 (5.4)	3 (1.7)	12.7
General Hospital, Minna	401 (9.1)	27 (15.4)	67.3
Ahmadu Bello University Teaching Hospital, Zaria	281 (6.4)	11 (6.3)	39.1
Adeoyo Maternity Hospital, Ibadan	945 (21.4)	38 (21.7)	40.2
Central Hospital, Benin City	1067 (24.2)	48 (27.4)	45.0
State Hospital, Ijaye, Abeokuta	174 (3.9)	10 (5.7)	57.5
Central Hospital, Warri	533 (12.1)	10 (5.7)	18.8
Total	4416	175	39.6

### All births, stillbirths and maternal and fetal characteristics

The prevalence of all births and stillbirths by the selected maternal and fetal characteristics is presented in Table 2. The mean age of the mothers was 28.6 ± 5.5; almost half of the births were by mothers who were self-employed; while a third (30.5%) was by unemployed mothers. Most births were by mothers resident in urban areas (82.8%), and the married (97.7%). More than one-third (39.7%) of the births were by mothers who were not booked for antenatal care, while 8.7% of them were referred to the health facility from home, traditional births attendants (TBAs) and other health facilities. Parity ranged from 0 to 13 and many (37.5%) of the births was by multiparous mothers. Only 5.2% of the births were by mothers who had stillbirths in the past. The majority of the babies weighed 2.50–3.99 kg at birth and 82.2% were born at gestational age of 37 weeks or more. Most of the births (86.8%) were by normal vaginal delivery; 11.5% were by caesarean section; while only 1.7% involved instrumentation.

**Table 2:** Percent Distribution of all Births and Stillbirths by maternal and fetal characteristics

	Number of births (%)		
Characteristic	Live birth	Stillbirth	Total
Maternal age			
≤ 19	136 (3.3)	9 (5.2)	145 (3.3)
20–24	877 (21.1)	33 (19.1)	910 (21.0)
25–29	1337 (32.2)	42 (24.3)	1379 (31.9)
30–34	1143 (27.5)	47 (27.2)	1190 (27.5)
35–49	662 (15.9)	42 (24.3)	704 (16.3)
Mean (Standard deviation) 28.6 (5.5)			
Employment Status			
Employed	495 (15.5)	18 (12.3)	513 (15.4)
Self employed	1586 (49.8)	69 (47.3)	1655 (49.7)
Unemployed	961 (30.2)	55 (37.7)	1016 (30.5)
Student	143 (4.5)	4 (2.7)	147 (4.4)
Place of residence			
Urban	2906 (82.7)	132 (82.5)	3038 (82.8)
Rural	605 (17.2)	28 (17.5)	633 (17.2)
Marital Status			
Single mothers	79 (2.3)	4 (2.5)	83 (2.3)
Married	3416 (97.7)	158 (97.5)	3574 (97.7)
Booked for Antenatal			
Not Booked	1466 (38.7)	101 (64.3)	1567 (39.7)
Booked	2320 (61.3)	56 (35.7)	2376 (60.3)
Referral			
Not referred	3559 (91.9)	119 (75.8)	3678 (91.3)
Referred	312 (8.1)	38 (24.2)	350 (8.7)
Parity			
0	1271 (30.8)	38 (21.9)	1309 (30.5)
1	995 (24.1)	31 (17.9)	1026 (23.9)
2–4	1540 (37.4)	72 (41.6)	1612 (37.5)
5–13	316 (7.7)	32 (18.5)	348 (8.1)
No of past stillbirths			
0	3311 (95.8)	112 (73.7)	3423 (94.8)
1+	146 (4.2)	40 (26.3)	186 (5.2)
Complications in any past pregnancy			
Yes	237 (7.1)	20 (14.6)	257 (7.4)
No	3085 (92.9)	117 (85.4)	3202 (92.6)
Birth weight			
< 1.50 kg	30 (0.7)	17 (10.9)	47 (1.1)
1.50–2.49 kg	329 (8.1)	42 (26.9)	371 (8.8)
2.50–3.99 kg	3539 (87.1)	85 (54.5)	3634 (85.9)
≥ 4.00 kg	165 (4.1)	12 (7.7)	177 (4.2)
Gestation age at delivery			
< 37 weeks	433 (16.6)	54 (44.3)	487 (17.8)
≥ 37 weeks	2179 (83.4)	68 (55.7)	2247 (82.2)
Mode of Delivery			
Normal Vaginal	3288 (87.2)	119 (78.8)	3407 (86.8)
Caesarean section	429 (11.4)	23 (15.2)	452 (11.5)
Others	56 (1.5)	9 (5.9)	65 (1.7)

The distribution of stillbirths by age indicates a similar proportion across ages 25–49; while younger women less than 20 years old experienced 5.2% of all stillbirths in the facilities. Stillbirth was highly prevalent among the self-employed (47.3%) and unemployed mothers (37.7%). Variation in the prevalence of stillbirth was observed by place of residence with 82.5% of all stillbirths recorded for mothers who resided in urban areas. Only 2.5% of all stillbirths were recorded for mothers who were single (never married, widowed, divorced or separated).

Stillbirth was more likely to occur among multiparous (2–4 deliveries) mothers. Women who had stillbirth were mainly unbooked (64.3%), not referred (75.8%), and had no history of previous stillbirths (73.7%). Many of the stillbirths had normal birth weight (2.50–3.99 kg), 7.7% were macrosomic and 37% low birth weight (≤2.49 kg). The majority were delivered at term (55.7%) and by spontaneous vaginal delivery (78.8%). Those delivered by caesarean section were 15.2%.

### Causes of stillbirth.

The causes of stillbirth were disaggregated by antepartum (macerated), intrapartum (fresh), and time of stillbirth unknown (Table 3). Antepartum (macerated) constituted 22% of the stillbirths and a major cause was maternal hypertensive disease. Other causes were antepartum haemorrhage, fetal growth retardation, antepartum unexplained, maternal infections, antepartum specific others such as cardiovascular disease, and severe congenital malformations. Intrapartum (fresh stillbirths) constituted 47% of the recorded stillbirths, which was mainly due to hypoxia. Other causes were pre-term labour and infections, while a substantial number were unexplained, and a few due to specific causes such as birth trauma. In general, hypoxia was a cause of 32.6% of all the stillbirths. Other major causes were maternal hypertensive disease (6.9%), and intrapartum unexplained (5.7%).

**Table 3:** Causes of stillbirth at time of death

Cause	Number of stillbirths (%) *N* = 175
ANTEPARTUM (MACERATED)	**39 (22.3)**
Congenital	1 (0.6)
Antepartum haemorrhage	5 (2.9)
Maternal hypertensive disease	12 (6.9)
Maternal infections	1 (0.6)
Antepartum specific others e.g. cardiovascular disease, etc.	1 (0.6)
Fetal growth retardation	5 (2.9)
Antepartum unexplained	2 (1.1)
Inadequate information	12 (6.9)
INTRAPARTUM (FRESH)	**83 (47.4)**
Congenital	0 (0.0)
Intrapartum hypoxia (acute event)	57 (32.6)
Preterm labor	1 (0.6)
Intrapartum infection	2 (1.1)
Intrapartum specific other**	5 (2.9)
Intrapartum unexplained	10 (5.7)
Inadequate information	8 (4.6)
TIME OF STILLBIRTH UNKNOWN	**53 (30.3)**

### Maternal and fetal factors associated with stillbirths

Results of the unadjusted and adjusted logistic regression models are presented in Table 4.

**Table 4:** Logistic Regression predicting the association between stillbirth and characteristics of mothers and newborn

Variable	Model 1 Unadjusted Odds Ratio (OR) (95% CI)	Model 2 Adjusted Odds Ratio (AOR) (95% CI)
Age		
≤ 19 (RC)	1.00	1.00
20–24	0.56 (0.26–1.21)	1.19 (0.25–5.51)
25–29	0.47 (0.22–0.99)*	0.62 (0.12–3.17)
30–34	0.62 (0.29–1.29)	0.80 (0.15–4.27)
35–49	0.95 (0.45–2.01)	0.62 (0.10–3.65)
Booked for antenatal		
No (RC)	1.00	1.00
Yes	0.35 (0.25–0.48)***	0.61 (0.29–1.26)
Referral		
Not referred (RC)	1.00	1.00
Referred	3.64 (2.48–5.34)***	3.74 (1.65–8.49)**
Parity		
0 (RC)	1.00	1.00
1	1.04 (0.64–1.68)	1.46 (0.60–3.52)
2–4	1.56 (1.04–2.33)*	3.07 (1.26–7.45)*
5+	3.38 (2.08–5.50)***	4.45 (1.20–16.50*
No of past stillbirths		
0 (RC)	1.00	1.00
1+	8.09 (5.44–12.0)***	3.79 (1.60–9.00)**
Complications in past pregnancy		
Yes (RC)	1.00	1.00
No	0.44 (0.27–0.73)**	1.02 (0.35–2.97)
Birth weight		
< 1.50 kg (RC)	1.00	1.00
1.50–2.49 kg	0.22 (0.11–0.44)***	0.47 (0.14–1.59)
2.50–3.99 kg	0.04 (0.02–0.07)***	0.18 (0.05–0.65)**
≥ 4.00 kg	0.12 (0.05–0.29)***	0.24 (0.04–1.31)
Gestation age at delivery		
< 37 weeks (RC)	1.00	1.00
≥ 37 weeks	0.25 (0.17–0.36)***	0.33 (0.16–0.67)**
Delivery mode		
Normal vaginal (RC)	1.00	1.00
Caesarean section	1.48 (0.93–2.34)	0.44 (0.16–1.17)
Others	4.44 (2.14–9.18)***	4.58 (1.41–14.8)*
Facility		
AKTH (RC)	1.00	1.00
KGH	0.34 (0.10–1.13)	0.80 (0.215–4.13)
GHM	1.93 (1.12–3.32)*	3.43 (0.71–16.4)
ABTH	1.09 (0.53–2.22)	0.54 (0.13–2.28)
AMH	1.12 (0.68–1.84)	1.47 (0.50–4.31)
CHB	1.26 (0.78–2.02)	1.00 (0.30–3.30)
SHI	1.63 (0.77–3.42)	1.79 (0.48–6.55)
CHW	0.51 (0.24–1.06)	0.30 s (0.02–3.31)

Note: ***p < 0.001; **p < 0.01; *p < 0.05; *OR* Odds Ratio, *CI* Confidence Interval

### Unadjusted model

Maternal age was of statistical significance only for mothers aged 25–29 who were 53% less likely than mothers less than 20 years to have stillbirths. Relative to mothers who were not booked for antenatal care in the facilities, mothers who booked for antenatal care were less likely to have stillbirth (OR 0.35 *p* < 0.001). The odds of stillbirth was significantly higher for mothers who were referred to the health facilities from home, TBA and other facilities than for those who were not referred (OR 3.64 *p* < 0.001). There was an increased likelihood of stillbirth for mothers who have had 2–4 deliveries (OR 1.56 *p* < 0.05) and 5 or more deliveries (OR 3.38 *p* < 0.001) compared with nulliparous mothers. Having had 1 or more stillbirths in the past increased the odds of stillbirth in the index pregnancy (OR 8.09 *p* < 0.001). Stillbirth was significantly lower among mothers who did not experience complications in any past pregnancy than those who did (OR 0.33 *p* < 0.01). The odds of stillbirth was lower when the fetal birth weight was 1.50–2.49 kg (OR 0.22 *p* < 0.001) and 2.50–3.99 kg (OR 0.04 *p* < 0.001), and ≥ 4.00 kg (OR 0.12 *p* < 0.001) relative to birth weight of < 1.50 kg. Compared with less than 37 weeks, gestational age of 37 weeks or more predicted less likelihood of stillbirth (OR 0.25 *p* < 0.001). Mothers whose mode of delivery was vaginal breach, ventouse, forceps and others were more likely to have stillbirths than those whose mode of delivery was normal vaginal (OR 4.44 *p* < 0.001). With respect to the facilities, stillbirth was more likely in General Hospital Minna than in Aminu Kano Teaching Hospital, Kano (OR 1.93 *p* < 0.05).

### Adjusted model

In the adjusted model, mothers who were referred to the facilities were significantly more likely to have stillbirth (AOR 3.74 *p* < 0.01). Stillbirth remained more likely among mothers who have had 2–4 and 5 or more deliveries. Past experience of stillbirth predicted a higher likelihood of having a stillbirth in the index pregnancy (AOR 3.79 *p* < 0.01). Births that weighed 2.40–3.99 kg were 82% less likely to be stillbirth compared to births that weighed < 1.50 kg. Babies delivered at thirty-seven or more weeks gestational age was associated with a lower likelihood of stillbirth than births of less than 37 weeks gestational age (AOR 0.33 *p* < 0.01). Delivery by other modes except caesarean section was significantly more likely to be associated with stillbirth than normal vaginal delivery (AOR 4.58 *p* < 0.05).

## Discussion

The study was designed to investigate the rate of stillbirths in eight referral hospitals in four out of the six geo-political zones of Nigeria, and to identify associated obstetric causes and socio-demographic factors. The results showed a mean stillbirth rate of 39.6 per 1000 births in the 8 hospitals that is slightly less than the national average [4]. However, two General hospitals in the north-central and south-west regions had stillbirth rates in excess of the reported national average. Thus, the results indicate a persisting high rate of stillbirths in referral hospitals without evidence of any substantial regional variation. Indeed, after adjusting for confounding variables, the rates did not differ significantly between the eight hospitals. This suggests that efforts to reduce the rate of stillbirths in the country should focus on all regions of the country, rather than on specific regions.

An assessment of the obstetric and clinical causes of death showed that more stillbirths occurred at the time of delivery (fresh stillbirths) than in the antepartum period (macerated stillbirths). An estimated 22% of the deaths were macerated compared to 47% of deaths that occurred during the intrapartum period. The large number of fresh stillbirths occurring at the time of delivery is worrisome as it indicates that the timing and method of delivery of the babies may not have followed standard obstetric practices. Indeed, the fact that a large proportion of the women were referred on emergencies during the intrapartum period after they had attempted to deliver in non-orthodox places of delivery (homes, traditional birth attendants, etc.) may have accounted for this. Efforts devoted to ensuring that women choose health facilities as their primary source of delivery rather than non-certified outlets would help to overcome this bottleneck.

Similarly, a large proportion of the macerated stillbirths were due to maternal obstetric complications – pregnancy hypertension, fetal growth retardation and maternal infections. This suggests that the high rate of pregnancy related complications among the women which are left unattended during the antenatal period predispose to these stillbirths before the time of delivery.

Among the associated factors for stillbirths, the results identified non-booking for antenatal care, referral from TBAs and faith-based clinics for emergency care, multiparity, previous experience of stillbirths, experience of obstetric complications in previous pregnancy, birth weight less than 1.50 kg, pregnancy less than 37 weeks gestation, and delivery by others means aside from normal vaginal and caesarean section as increasing the likelihood of stillbirths in the hospitals. However, after adjusting for confounders in the full logistic regression model, referral from TBAs and other non-facilities sources of care, multiparity, previous experiences of stillbirths, low birth weight, gestational age at delivery and mode of delivery remained significant predictors of the likelihood of stillbirth.

These results confirm the importance of antenatal care for identifying pregnancy complications and taking prompt action to manage the complications that increase the risk of stillbirths. As preventing stillbirths becomes a more visible goal of the maternal and child health agenda, attention to the important role of antenatal care in reducing stillbirths becomes critical [17, 18]. The results also indicate the need for steps to be taken to ensure that women are educated about the importance of delivering in health facilities rather than at home or in non-orthodox places such as in the homes of traditional birth attendants or in Churches. The current situation where only about 33% of pregnant women are attended to at birth by Skilled Birth Attendants (doctors, nurses and midwives) [24] is worrisome, and accounts for the high rate of preventable fresh stillbirths reported in this study. Clearly, increasing women’s access to skilled delivery care is an important measure to reduce the high rate of stillbirths in the country.

The finding that multiparity, previous experiences of stillbirths, pre-term delivery and complications in any previous pregnancy are significantly associated with increased odds of stillbirth adds more credence to the importance of antenatal care. These conditions can be managed effectively and prevented when women attend antenatal care regularly, with the effective delivery of quality antenatal care that can reduce the risk of severe complications leading to stillbirths. The present tendency for pregnant women in Nigeria not to receive antenatal care and to turn up only at delivery or at the time of emergency obstetric care is worrisome [19–21]. The lack of antenatal care has turned up repeatedly as a risk factor for maternal mortality [22–26], and now as a predictor of the high rate of stillbirths in the country. Clearly, increasing women’s access to evidence-based and quality antenatal care must be given priority policy attention in policies to improve maternal health and reduce the stillbirth rate in the country.

Of interest was the finding in the logistic regression model, which showed that women with instrumental delivery (vacuum extraction and forceps delivery) were four times more likely to experience stillbirths as compared to those delivering vaginally. The caesarean section rate in this study of 11.5% was within the recommended rate of caesarean section recently set by the World Health Organization [27]. However, the increased stillbirth rate in women with instrumental delivery after adjusting for confounding variables suggests either poor skills in instrumental delivery or that women who ought to deliver by caesarean section were being delivered by instrumental delivery with resultant negative outcomes for fetal viability. The study therefore confirms previous reports which suggest the under-utilization of caesarean sections in many sub-Saharan African countries [28, 29] needed to address the burden of maternal ill-health. Although there is evidence to suggest that women are averse to caesarean delivery in Nigeria [30, 31], strategic public health education can help to convince women and care-givers that such mode of delivery is to save the lives of the women and their babies.

The major strength of this study is its multi-centre design and the involvement of multiple hospitals in four out of the six geo-political zones in the country. This has allowed regional comparison of the results, permitting its generalization to the wider Nigerian health systems context given that no statistically significant difference was found in rates between the hospitals when all factors were adjusted. The results are not only useful for preventing stillbirths within the participating health facilities; it also has implications for the development of policies for the improvement of maternal health care and the reduction of the high rate of stillbirths at a health systems level in the country.

By contrast, because this study is facility-based, the reported stillbirth rates and determinants exclude stillbirths that occur in births outside facilities. The major limitation of the study is its retrospective design and the fact that all cases of stillbirths may not have been captured due to poor record keeping in the hospitals. For example, the causes of up to 30% of stillbirths could not be determined due to inadequate record keeping. However, we made specific and rigorous efforts to ensure accurate data collection in the eight participating hospitals. We deliberately chose a retrospective design since this was part of a larger study whose objective was to assess the existing quality of emergency obstetric care in the referral hospitals, with the goal being to design appropriate interventions for addressing the identified bottlenecks in service delivery. A prospectively designed study would have compromised our ability to obtain accurate information on the state of delivery of maternal health care in the hospitals.

The importance of the need to ensure accurate data collection informed our concentration of data collection in the immediate preceding 6 months of the study in order to reduce the potential for data mis-handling. In particular, we used multiple approaches to identify all cases of stillbirths in the hospitals, including examination of records in the maternity wards, record keeping departments and in the delivery suites. Only when these multiple sources of record keeping were in agreement were the cases of stillbirths accepted as true. Also, the record of early neonatal deaths was separate from stillbirths in all the facilities, indicating accurate assessment of stillbirths. Thus, we believe that the results are accurate and represent the current state of available data on stillbirths in the referral hospitals.

## Conclusion

We conclude that the independent predictors of high stillbirth rates in Nigeria’s referral hospitals include unbooked status and late referrals, multiparity, previous experience of stillbirths, experience of pregnancy complications, low birth weight, delivery at less than 37 weeks gestation and the use of instrumental vaginal delivery. Efforts to address these bottlenecks should include increasing women’s access to skilled pregnancy care and caesarean delivery when needed, the improvement of maternal health care in secondary and tertiary care facilities, and public health education so that women can seek appropriate and immediate evidence-based and skilled pregnancy and delivery care. We believe that these reforms should be based on a systematic strengthening of the health care system in the country, so it can respond appropriately and effectively to the needs of pregnant women seeking care in referral facilities.

## Data Availability

The dataset analyzed for the current study is available from the corresponding author on request.

## Abbreviations

AOR: Unadjusted Odds Ratio
CHERG: Health Epidemiology Reference Group
CI: Confidence Interval
GAPPS: Global Alliance for Prevention of Prematurity and Stillbirths
ICMJE: International Committee of Medical Journal Editors
NHREC: National Health Research Ethics Committee
OR: Odds Ratio
PHC: Primary Health Care
TBA: Traditional Births Attendant
WHO: World Health Organization
